# Sublingual Immunotherapy: A Useful Tool for the Allergist in Private Practice

**DOI:** 10.1155/2016/9323804

**Published:** 2016-05-31

**Authors:** Diego Saporta

**Affiliations:** Private Practice, 470 North Avenue, Elizabeth, NJ 07208, USA

## Abstract

This is a review of the author's experience with Sublingual Immunotherapy in a private office setting. Sublingual Immunotherapy should be considered by any allergy practitioner as a useful tool. Sublingual Immunotherapy is safe while at the same time it is effective. It enables the practitioner to treat asthmatics and young children without the concerns implicit with allergy injections.

## 1. Introduction

Sublingual Immunotherapy (SLIT) has been used in the US since the late 19th century. The earliest description dates back to the year 1900 [1]. In the last 30 years, good quality research allowed this treatment modality to be clearly considered as a useful, effective, and safe alternative for the administration of immunotherapy [2–4]. Well-designed studies and review studies confirm SLIT's safety and effectiveness [5–8].

Safety is one of the striking features of SLIT. It is a well-known fact that Subcutaneous Injection Immunotherapy (SCIT) can elicit reactions as either local arm reactions or systemic reactions, ranging from mild to severe [9–13]. There are cases of mortality reported in the literature after SCIT administration and even though the frequency of mortality cases appears to be decreasing over time [14, 15] the spectrum of this possibility is ever present to any practitioner that administers injectable immunotherapy.

SLIT is not problem free. There are reactions after SLIT administration, usually known as Adverse Events (AEs). Published literature commonly describes these reactions as mild and sometimes as self-resolving [16]. In most of the series, the incidence of these AEs is relatively small [17, 18]. There are no mortality cases related to SLIT administration [16, 19, 20]. Only a few cases of severe reactions after SLIT administration have been reported, where patients developed asthma attacks requiring hospital care [21].

Reports of SLIT using few or several allergens are infrequent [18, 22, 23]. Most of the reports about SLIT are based on its administration for monosensitized patients.

## 2. SLIT in a Private Practice Setting

The author will summarize his experience with SLIT, which was successfully incorporated into a private practice setting in 2003. The author was trained at the American Academy of Otolaryngic Allergy (AAOA) where he learned to use the intradermal test with multiple serial dilutions (Intradermal Dilutional Test or IDT) [24, 25]. The advantage of this test is that stronger concentrations of the allergen can be injected allowing the identification of patients that react only to a high concentration of the antigen (a test with similar characteristics is the Modified Quantitative Test or MQT which is also taught at the AAOA and the Pan American Allergy Society). The IDT is the most commonly used test by the author for the diagnosis and management of allergic conditions. With this test it is usual to identify many reactive allergens. It is rare to find a patient reactive to only 2 or 3 allergens. Like other practitioners trained with this technique, the author has been handling multiple allergens since the beginning of his allergy practice so that injectable vials and SLIT bottles are commonly mixed with multiple allergens [20, 26]. These allergens are obtained from the major allergen manufacturing companies therefore assuring quality and allergen standardization when available.

The author developed a protocol for SLIT administration based on 1 : 5 dilutions [27]. The drops are administered daily; dose is slowly increased once a week from 1 drop per day up to 5 drops per day over a 5-week period. When a bottle is finished a new bottle 5 times more concentrated is mixed. Escalation continues without the need to retest the patient. When the maintenance dose is attained treatment will continue to complete 3–5 years, the same as with SCIT. Drops are held under the tongue for 20–30 seconds and then swallowed. With this protocol a severe or serious AE has never been encountered despite many hundreds of patients being treated.

The author has performed a series of studies in the setting of his private practice. These studies do not meet the same standards of a study from an academic institution. Their objective was to prove over time that the protocol being used was mostly in agreement with existing published literature on SLIT. Through those studies it was possible to establish that the treatment results obtained on patients treated using this protocol were positive, proving the technique effective and in agreement with data from the European literature. In other words, the clinical impression that the protocol provided effective and safe treatment was documented, and patients in a private office setting were able to benefit from a treatment modality often found only in clinics.

### 2.1. Evidence for Efficacy

The first evaluation included a group of patients that received SLIT, but roughly half of those patients had been previously treated with SCIT and for a variety of reasons changed into SLIT [27]. Overall 90% of the patients expressed satisfaction with SLIT. Of the patients previously treated with SCIT, 75% of the patients found both treatments similar, and 8% thought SCIT was better, but interestingly 17% thought SLIT was better.

The second evaluation was a head-to-head comparison between SCIT and SLIT [28]. This study included only patients that were treated with either modality. The improvement was measured by a decrease in the symptom-score and medication use and by an increase in the Peak Flow (PF) value. Both groups exhibited a symptomatic improvement and a decrease in the use of medications, with an increase in the PF value. The parameters analyzed attained statistical significance with either modality but the intergroup comparison did not yield significant differences. The conclusion from this observation was that SLIT performed similar to SCIT from the clinical point of view.

### 2.2. Evidence for Safety

While a severe reaction never occurred after administration of SLIT with this protocol, AEs during SLIT administration are not uncommon. The AEs usually reported in the literature include labial or buccolingual edema, itching in oral cavity or other parts of the face, throat irritation, rhinoconjunctivitis, and gastrointestinal (GI) symptoms [16, 19, 29, 30].

In the author's experience [27] 12% of the patients reported an AE, half of which involved the skin which is in agreement with the reported literature. It has been suggested that severity and/or persistence of the reaction can lead to treatment discontinuation [31].

There is still no universally accepted system to grade and classify AEs. It has been proposed to classify AEs according to site of origin as local versus systemic [31] or according to severity as mild, moderate, and severe [31] or as mild, moderate, and serious [6].

Local reactions are reported as occurring frequently; systemic reactions are reported as occurring rarely. There is no uniform criterion for inclusion of symptoms. For example, local reactions are reported as oral itching and/or swelling and as GI complaints (nausea, stomach pain, vomiting, abdominal pain, and diarrhea) but also as altered taste perception, itching of lips, swelling of lips, itching of oral mucosa, swelling of oral mucosa, swelling of tongue, glossodynia, mouth or tongue ulceration, throat irritation, or uvular edema [31, 32].

Systemic reactions are reported as cutaneous symptoms (urticaria and itching), ocular symptoms (redness, itching, and tearing), nasal symptoms (itching, sneezing, rhinorrhea, and obstruction), asthma symptoms (cough, wheezing, shortness of breath, and chest tightness) [31], or rhinitis, asthma, urticaria, angioedema, and hypotension [32]. These reactions are usually reported as mild. Their incidence is similar in active or placebo groups except for oral and gastrointestinal reactions which appear to be more frequent in active groups, regardless of age [17, 33, 34].

AEs usually occur during the induction phase and with low doses of allergen [6, 29, 32] and are reported to gradually decrease as treatment progresses [31]. Gastrointestinal complaints are reported to occur more frequently with higher doses [32].

AE management usually involves dose adjustment or symptomatic treatment [6, 17, 34, 35]. Some reports suggest that treatment discontinuation because of AEs is not high [36] but in a clinical setting discontinuation rate despite symptomatic improvement has been reported as 31%, apparently related to the development of local reactions [29].

The author collected all the AEs that occurred in his practice during a 5-year period [37]. During that period 62 patients reported 39 different symptoms a total of 109 times. No AE was serious or severe. In agreement with published literature most of the AEs events involved the skin, the oropharynx, and the GI system. Defining a complete course of therapy as 3 years, it was found that only 14.5% of the 62 patients that developed AEs completed the treatment. In other words, it became clear that 85.5% of patients that developed an AE during SLIT administration quit treatment. Even more, 37.1% had quit soon after onset of the AE (usually within 3 months of treatment initiation). This report appears to be the only one that clearly suggests that an AE during SLIT administration, even if not severe, will be significant enough for the patient to terminate treatment.

To further assess this finding, an additional 100 charts of SLIT patients were randomly evaluated and it was found that there was a discontinuation rate, not necessarily related to AEs, of 27%–34%. When comparing the data from the group with AEs (85.5% discontinuation) versus the group with spontaneous discontinuation of treatment not necessarily related to an AE (27%–34%), the importance of the AE as a factor to determine treatment discontinuation becomes clear.

In the same study [37] 15/62 patients reported GI symptoms. Of these, 14/15 or 93.3% had quit at the time of data collection. While the difference lacked statistical significance probably related to sample size, the different percentages may suggest that patients that develop GI symptoms are even more likely to quit.

SLIT literature generally analyzes what percentage of treated patients get AEs and what the AEs are. If the information we found could be confirmed, then it is to be expected that any patient who develops an AE during SLIT administration would be likely to quit treatment, even more so if the AE involved the GI system. This is an important piece of information for the practitioner in private practice. By anticipating the probable outcome of a patient that develops an AE during SLIT administration, the treating physician can intervene before the patient quits.

AEs involving the GI system are reported rather frequently and they are considered by some researchers as local reactions [31, 32] and reported to be a cause for treatment discontinuation [32].

Reports regarding the use of sublingual tablets describe many AEs but GI symptoms are not usually reported [38–41]. On the other hand, a study that used a liquid form, equivalent to the Ragweed tablet [42], did report GI side effects. As the only difference between a bottle of SLIT and the allergy tablet is that the tablet lacks the glycerin used as diluent in the SLIT bottles, the author suspects that the GI AEs are a consequence of the glycerin and not of the allergens.

In support of this contention the author observed the following:

- When patients had AEs not related to the GI system, the AEs would abate by diluting the treatment bottle, but this would not happen when the AE involved the GI system. The dilution process implies administering a lesser amount of allergen (less concentrated) but the diluent concentration (glycerin) remains the same.
- The GI AE would often get worse with continued administration of SLIT. This observation has also been suggested in the literature, as GI AEs are reported to be dose related. In other words, they occur more frequently with higher doses [32, 35].
- Diluting the bottle in saline sometimes led to a resolution of the GI complaint. We observed that this only worked for diluted allergens at the beginning of the treatment. As the dose advanced and the concentration of the allergens increased, so did the content of glycerin and the AE would reappear.

### 2.3. Advantages of SLIT for the Administration of Immunotherapy

As SLIT is effective and extremely safe it can be considered the ideal modality for home-based immunotherapy. It can be used when patients are not good candidates for SCIT as is the case with young children, very old patients, and high-risk patients. There is a subgroup of patients, not necessarily children, who are scared of needles. For these patients oral vaccines are ideal.

With SLIT there is no need for treatment interruption for vacations or relocations. If SLIT treatment is interrupted it is very easy to restart. Glycerin is a potent protein stabilizer. When glycerin is used as the diluent for mixing SLIT, potency of the allergens is maintained for a long time; therefore these drops do not need refrigeration.

SLIT implies a noninjectable route; therefore local arm reactions are nonexistent. These are sometimes painful and often interfere with injectable dose advancement.

SLIT may offer an economical advantage: patients with no medical insurance coverage or those that have insurance but with high copays can easily be treated with SLIT instead of SCIT. The patient also saves time as there is no need to come to the office, obviating the need to wait 30 minutes after injections.

These factors imply a potential for more compliance. At least one report suggests that adherence to SLIT could be more than 90% [43]. When age, health, physical location, or economics interfere with transportation to the office, SLIT should be considered. There is also a role for SLIT in special circumstances where SCIT can be problematic or even controversial, like when treating very young, asthmatic, or pregnant patients.

### 2.4. Use of SLIT in Asthma and Pregnancy

The asthmatic patient and the very young patient are difficult to manage with specific immunotherapy, even more so if the patient is an asthmatic child. These difficult-to-treat patients can safely receive SLIT [44].

Guidelines for the administration of injectable immunotherapy do not advise treating patients younger than 5 years, as reactions, if they were to occur, are more difficult to manage [45]. It is known that patients with nasal allergies, if left untreated, have a 19% chance of developing asthma [46] but early administration of immunotherapy will prevent the development of new sensitivities, will improve asthma if present at the time of treatment initiation, and will prevent the development of asthma in the future [47–49] so it is only logical to provide specific immunotherapy as soon as possible to stabilize and revert the inflammation that eventually leads to asthma. SLIT has successfully been used in very young children [50].

Young children are routinely seen at the author's office. A brief review of treatment results in a small group of 10 children with nasal allergies and asthma was done [51]. The study confirmed that SLIT was not only effective in treating childhood asthma but also safe for these difficult-to-treat patients. An interesting observation of this small series was that, a few months after initiation of therapy, the children that were on inhaled medication were not using inhalers anymore. It is the ongoing clinical observation of the author that patients on inhalers can stop using them earlier than allergy medication.

In our office, the treatment results are evaluated using a symptom-scoring sheet that is filled out by nursing personnel every time a new vial for SCIT or a bottle for SLIT is started. The scoring sheet includes symptoms, information on medication use, and an objective evaluation consisting of the use of a Peak Flow Meter to assess the value of the PF [52]. It was determined that when immunotherapy was successful, the PF value increased even if the patient was not asthmatic. This observation gives support to the concept of the Unified Airway [53].

From the analysis of the scoring sheets it was realized that patients often deny having asthma despite having one or more of the symptoms characteristic of lower airway inflammation or even if using an inhaler prescribed by their primary care doctor. This is an important concept as severe reactions following allergen injections for testing or treatment are more common in asthmatics [14, 54, 55]. Therefore identification of patients with potential involvement of the lower airway is important. These patients should be tested with more precautions, and treatment should be done, if possible, with SLIT.

The treatment of the pregnant patient is problematic. Present guidelines do not suggest advancing the injectable dose in a pregnant patient. There is not much information about the administration of SLIT during pregnancy but a retrospective chart review and a prospective report suggested that it was safe to administer SLIT during pregnancy even though the numbers in those papers were small for definite conclusions [56, 57].

The success of the treatment protocol is strongly based on it being safe and effective. It is also important in order to increase compliance that the protocol is easy for the patient to follow as this is a home-based therapy. Many protocols advocate leaving the drops under the tongue for several minutes. The author advises the patients to keep the drops under the tongue for 20–30 seconds before swallowing them. This is based on evidence from pharmacokinetic studies that upon a brief contact with the sublingual mucosa the allergen attaches to it and the attachment lasts for hours [58, 59].

It is common in a private practice setting that the patient on immunotherapy leaves for a long vacation, changes jobs, moves far from the office, or loses insurance coverage. In all these circumstances it may be necessary to change treatment modalities “back and forth” from SCIT into SLIT or vice versa. Because of SLIT's safety it is very easy to change from SCIT into SLIT, which is very important in order to keep the patient treated while circumstances change. Changing from SLIT into SCIT may not be safe. The author decreases the administered dose often “going back” to the initial formulation when changing from SLIT into SCIT.

### 2.5. Future of SLIT

SLIT in the US is not approved by the FDA. Its use implies an off-label use of the allergenic extracts. Still there is a growing interest in this treatment modality and yearly courses on SLIT are offered by the main allergy academies. The situation is different in Europe, where an estimated 45% to 80% of all immunotherapy is administered as SLIT [60].

The author anticipates that the use of SLIT in the US for the private setting will only increase in the near future for 2 reasons:

 (a) Present day medical insurances are becoming very expensive, with high deductibles and high copays. For this reason, many patients will not be able to afford the lengthy allergy treatment with weekly injections. Although SLIT is an out-of-pocket expense it is becoming a more viable option in those circumstances. It also saves the patient time and transportation costs. From the practical point of view it is observed that there are patients that find the cost of the noncovered service (SLIT) preferable to the covered service (SCIT).
 (b) Prevalence of asthma (and allergic rhinitis) has shown a worldwide increase over the last two to three decades [61]. The increase appears to be greater in children and young adults (asthma affects 20%–25% of the total population but 20%–40% of childhood population) [62].
 After severe storms affected the author's geographical area it was observed that the patient population consulting at the author's office for allergic conditions appeared to be younger, more sensitive, and more reactive [63]. It is likely that the same changes are happening in other areas of the US that have been flooded in the last decade. Because of safety concerns when treating children and asthmatic patients, even more if patients are more reactive, SLIT will likely acquire a more significant role in the management of their allergic conditions.

In 2014 the FDA approved allergy tablets for use in the US, for the treatment of pollen-induced allergic rhinitis with or without conjunctivitis [64], when there is a confirmation by skin or blood test that the patient reacts to the pollen(s) contained in such tablets. There are 3 such tablets, one that provides ragweed, one that provides timothy, and the third one which is a mixture of 5 grasses.

In this case, the allergen(s) is (are) delivered to the oral mucosa in a rapidly dissolving tablet. The patient is treated with a preset dose of the allergen(s) without an updosing schedule. Because ultimately the allergens are delivered to the oral mucosa in a similar way as with SLIT it could be speculated that FDA approval for these tablets will eventually ease the approval of the same allergens in a liquid form, mixed at doctors' offices.

## Additional Points

SLIT is a versatile treatment modality. Its safety makes it ideal for handling the difficult-to-treat patient like the asthmatic and the child. If SLIT were reimbursed by insurance companies it would potentially be the treatment of choice for those difficult-to-manage circumstances. It could also be more convenient in many other cases like when there are recurrent local arm reactions, the patient has time management problems that prevent coming to the office for shots, or the patient lives far away or has physical difficulties getting to and from the office.
